# GDF15 regulates Kv2.1-mediated outward K^+^ current through the Akt/mTOR signalling pathway in rat cerebellar granule cells

**DOI:** 10.1042/BJ20140155

**Published:** 2014-04-25

**Authors:** Chang-Ying Wang, An-Qi Huang, Meng-Hua Zhou, Yan-Ai Mei

**Affiliations:** *State Key Laboratory of Medical Neurobiology, School of Life Sciences and Institutes of Brain Science, Fudan University, Shanghai 200433, China

**Keywords:** Akt/mammalian target of rapamycin (mTOR), cerebellar granule neuron (CGN), delayed-rectifier outward K^+^ current (*I*_K_), growth/differentiation factor 15 (GDF15), Kv2.1, 4-AP, 4-aminopyridine, CGN, cerebellar granule neuron, CHX, cycloheximide, DIC, days in culture, DMEM, Dulbecco’s modified Eagle’s medium, ERK, extracellular-signal-regulated kinase, GAPDH, glyceraldehyde-3-phosphate dehydrogenase, GDF15, growth/differentiation factor 15, *I*_A_, fast-transient outward K^+^ current, *I*_K_, delayed-rectifier outward K^+^ current, MAPK, mitogen-activated protein kinase, MEK, MAPK/ERK kinase, MIC-1, macrophage inhibitory cytokine-1, mTOR, mammalian target of rapamycin, NP40, Nonidet P40, PI3K, phosphoinositide 3-kinase, PKA, protein kinase A, qPCR, quantitative real-time PCR, R-Smad, receptor-regulated Smad, TGFβ, transforming growth factor β, TGFβR, TGFβ receptor, TTX, tetrodotoxin

## Abstract

GDF15 (growth/differentiation factor 15), a novel member of the TGFβ (transforming growth factor β) superfamily, plays critical roles in the central and peripheral nervous systems, but the signal transduction pathways and receptor subtypes involved are not well understood. In the present paper, we report that GDF15 specifically increases the *I*_K_ (delayed-rectifier outward K^+^ current) in rat CGNs (cerebellar granule neurons) in time- and concentration-dependent manners. The GDF15-induced amplification of the *I*_K_ is mediated by the increased expression and reduced lysosome-dependent degradation of the Kv2.1 protein, the main α-subunit of the *I*_K_ channel. Exposure of CGNs to GDF15 markedly induced the phosphorylation of ERK (extracellular-signal-regulated kinase), Akt and mTOR (mammalian target of rapamycin), but the GDF15-induced *I*_K_ densities and increased expression of Kv2.1 were attenuated only by Akt and mTOR, and not ERK, inhibitors. Pharmacological inhibition of the Src-mediated phosphorylation of TGFβR2 (TGFβ receptor 2), not TGFβR1, abrogated the effect of GDF15 on *I*_K_ amplification and Kv2.1 induction. Immunoprecipitation assays showed that GDF15 increased the tyrosine phosphorylation of TGFβRII in the CGN lysate. The results of the present study reveal a novel regulation of Kv2.1 by GDF15 mediated through the TGFβRII-activated Akt/mTOR pathway, which is a previously uncharacterized Smad-independent mechanism of GDF15 signalling.

## Abstract 2

In the present paper, we report for the first time that GDF15 increases Kv2.1 expression through TGFβRII to activate the Akt/mTOR pathway, which reveals an unanticipated role of the non-Smad-dependent pathway in the previously uncharacterized signalling mediated by GDF15.

## INTRODUCTION

GDF15 [growth/differentiation factor 15; also known as MIC-1 (macrophage inhibitory cytokine-1)], is a novel member of the TGFβ (transforming growth factor β) superfamily that was discovered in a screen for the conserved consensus sequences of TGFβs [1]. GDF15 plays key roles in prenatal development and the regulation of cellular responses to stress signals, inflammation and tissue repair after acute injuries in adult life [2]. GDF15 has also been identified as a downstream target of p53, suggesting that it plays a role in the injury response to DNA damage and cancer [3]. Additionally, GDF15 is known as a novel cardioprotective cytokine that protects the heart from ischaemia/reperfusion injury [4]. However, the mechanism of GDF15 induction remains elusive.

A study of the nervous system indicated that GDF15 is widely distributed in the central and peripheral nervous systems, including the cortex, thalamus and striatum [5]. It has also been observed that GDF15 acts as a pro-survival and protective factor *in vitro* and *in vivo* for embryonic and lesioned dopaminergic neurons in the midbrain and the substantia nigra and is dramatically up-regulated in cryolesioned cortical neurons [6,7]. A report from Strelau et al. [8] showed that GDF15-deficient mice exhibit progressive postnatal losses of spinal, facial and trigeminal motoneurons, as well as sensory neurons in the dorsal root ganglia. Recently, one study suggested that GDF15 was a potential support factor for neuronal synaptic development and integration during axonal elongation [9]. Although these data suggest that GDF15 plays a critical role in the central nervous system, the signal transduction pathways and the receptor subtypes involved are not well understand.

CGNs (cerebellar granule neurons) are small glutamatergic cells that constitute the largest homogeneous neuronal population in the mammalian brain. Owing to their postnatal generation and the well-defined developmental pathway *in vitro*, primary cultures of rat CGNs have been established as a model for studying neuronal maturation, apoptosis, differentiation and synaptic plasticity [10]. Previous studies have indicated that growth and differentiation factors can either stimulate or inhibit CGN development and maturation by regulating multiple signalling pathways [11,12]. Moreover, GDF15 can induce neuroprotective effects that prevent the death of K^+^-deprived CGNs by activating Akt and inhibiting constitutively active ERK (extracellular signal-regulated kinase) [13].

One of the key activities during CGN development and maturation is the regulation of the expression of the K^+^ channels [11,12]. CGNs grown in primary culture display several voltage-activated outward K^+^ currents, including the *I*_A_ (fast-transient outward K^+^ current), *I*_K_ (delayed-rectifier outward K^+^ current), *I*_K(so)_ (non-inactivating K^+^ current) and *B*_K(Ca)_ (calcium-activated K^+^ current). We reported previously that the enhancements of the *I*_K_ and *I*_A_ were associated with CGN apoptosis induced by low K^+^/serum-free culture medium [14,15]. In the normal development state, however, increases in *I*_K_ and *I*_A_ improved the migration and maturation of CGNs [16,17]. Furthermore, TGFβ1, a member of the TGFβ family, promoted CGN maturation by increasing the expression of Kv2.1, the main α-subunit of the *I*_K_ channel, via a non-Smad pathway [12].

Therefore the initial objective of the present study was to examine whether the neuroprotective role of GDF15 involves the modulation of the K^+^ channels to prevent death in low K^+^/serum-free cultured CGNs or whether it involves its neurotrophic factor role in neurodevelopment and maturation. We unexpectedly found that GDF15 did not decrease the *I*_K_ or *I*_A_ amplitude or protein expression in low K^+^/serum-free cultured CGNs; in contrast, GDF15 significantly increased the *I*_K_ amplitude by enhancing the expression of the Kv2.1 α-subunit of the *I*_K_ channel in immature CGNs. The regulation of the GDF15-induced Kv2.1 expression, as well as the signalling pathways and the receptor associated with the effects of GDF15, was also investigated. The present study revealed, for the first time, that GDF15-mediated signalling modulates K^+^ channel expression for neuronal development and function, and identified the receptor involved in this function.

## EXPERIMENTAL

### Cell culture

All experimental procedures were carried out in accordance with the European Union guidelines for the care and use of laboratory animals (Council Directive 86/609/EEC). Cells were derived from the cerebella of 7-day-old Sprague–Dawley rat pups as described previously [18]. Isolated cells were plated on to 35 mm Petri dishes coated with 1 μg/ml poly-L-lysine at a density of 10^6^ cells/ml. Cultured cells were incubated at 37°C with 5% CO_2_ in DMEM (Dulbecco's modified Eagle's medium) supplemented with 10% FBS, 5 μg/ml insulin, 25 mM KCl and 1% antibiotic/antimycotic solution. After culture for 24 h, 5 μM cytosine β-D-arabinofuranoside was added to the culture medium to inhibit the proliferation of non-neuronal cells. The cells were used for experiments after 4–5 DIC (days in culture), unless otherwise indicated.

### Patch-clamp recordings

Whole-cell currents of the granule neurons were recorded using the conventional patch-clamp technique. All currents were recorded using a multiclamp 200B amplifier (Axon Instruments) operated in voltage-clamp mode. Data acquisition and analysis were performed using the pClamp 8.01 soft-ware (Axon Instruments) and/or the Origin 8 analysis software (Microcal Software). Prior to *I*_K_ recording, the culture medium was replaced with a bath solution containing 145 mM NaCl, 2.5 mM KCl, 10 mM Hepes (pH 7.4), 1 mM MgCl_2_, 1 μM TTX (tetrodotoxin), 5 mM 4-AP (4-aminopyridine) and 10 mM glucose. Soft-glass recording pipettes were filled with an internal solution containing 135 mM potassium gluconate, 10 mM KCl, 10 mM Hepes (pH 7.3), 1 mM CaCl_2_, 1 mM MgCl_2_, 10 mM EGTA, 1 mM ATP and 0.1 mM GTP. The pipette resistance was 4–6 MΩ after it was filled with internal solution. All recordings were performed at room temperature (23–25°C). The cultured granule cells selected for electrophysiological recording exhibited common morphological characteristics of healthy cells, such as fusiform soma with two main neuritis of similar size. Additionally, the mean capacitance of the recorded cells for the control group (9.17±0.23 pF) and for the GDF15-treatment group (9.36±0.21 pF) showed no significant difference, indicating that comparable granule cells were used in the experiments.

### Western blot analysis

The cells were lysed in Hepes/NP40 (Nonidet P40) lysis buffer [20 mM Hepes, 150 mM NaCl, 0.5% NP40, 10% glycerol, 2 mM EDTA, 100 μM Na_3_VO_4_, 50 mM NaF (pH 7.5) and 1% proteinase inhibitor cocktail] on ice for 30 min. After centrifugation (12000 ***g*** for 15 min at 4°C), the supernatant was mixed with 2× SDS loading buffer and boiled for 5 min. The proteins were separated by SDS/PAGE (10% gel) and were then transferred on to a PVDF membranes (Millipore). The membrane was blocked with 10% (w/v) non-fat dried skimmed milk powder and incubated at 4°C overnight with the following primary antibodies: mouse monoclonal antibody against Kv2.1 or Kv2.2 (1:1000 dilution; UC Davis, Davis, CA, U.S.A.), rabbit polyclonal antibody against phosphorylated insulin receptor β subunit (1:1000 dilution; Cell Signaling Technology), rabbit monoclonal antibody against phosphorylated ERK1/2 or total ERK1/2 (1:1000 dilution; Cell Signaling Technology), rabbit monoclonal antibody against phosphorylated Akt (1:2000 dilution; Cell Signaling Technology), rabbit polyclonal antibody against phosphorylated mTOR (mammalian target of rapamycin; 1:1000 dilution; Cell Signaling Technology), rabbit polyclonal antibody against TGFβR2 (TGFβ receptor 2; 1:500 dilution; Millipore), mouse monoclonal antibody against phosphorylated tyrosine (1:2000 dilution; Cell Signaling Technology), or mouse monoclonal antibody against GAPDH (glyceraldehyde-3-phosphate dehydrogenase; 1:10000 dilution; KangChen Bio-Tech). After extensive washing in TBST (TBS with 0.03% Tween 20), the membrane was incubated with horseradish peroxidase-conjugated anti-mouse or anti-rabbit IgG (1:10000 dilution; KangChen Bio-Tech) for 2 h at room temperature. Chemiluminescent signals were generated using a SuperSignal West Pico trial kit (Pierce) and were detected by exposure to X-ray film or using the ChemiDoc XRS System (Bio-Rad Laboratories). The Quantity One software (version 4.6.2; Bio-Rad Laboratories) was used for background subtraction and for the quantification of the immunoblot data.

### qPCR

To measure the Kv2.1 mRNA levels, qPCR (quantitative real-time PCR) analysis was performed with the primer sequences forward, 5′-ATTGCCGGGGTCCTGGTGATTG-3′ and reverse, 5′-GCCCTCTTGGTCCATTTCCACTTGTT-3′. To control for sampling errors, qPCR for the housekeeping gene cyclophilin D was performed with the primer sequences forward, 5′-GGCTCTTGAAATGGACCCTTC-3′ and reverse, 5′- CAGCCAATGCTTGATCATATTCTT-3′. The reaction solution contained 2.0 μl of diluted reverse transcription PCR product, 0.2 μM of each paired primer and Power SYBR Green PCR master mix (Toyobo). The annealing temperature was set at 62°C and 40 amplification cycles were used. The absolute mRNA levels in each sample were calculated according to a standard curve determined using serial dilutions of known amounts of specific templates plotted against the corresponding cycle threshold (*C*_T_) values. The normalized ratio of the target gene over cyclophilin D in each sample was calculated. The specificity of the primers was verified by both gel electrophoresis and sequencing of the PCR products.

### Immunoprecipitation

For the detection of tyrosine-phosphorylated TGFβR2, CGNs incubated with or without GDF15 for 5 min were lysed in Hepes/NP40 lysis buffer as described above. After centrifugation (12000 ***g*** for 15 min at 4°C), the cell lysate was mixed with a rabbit polyclonal antibody against TGFβR2 (1:500 dilution; Millipore) at 4°C. After overnight incubation, Protein A/G-conjugated (1:25 dilution; Santa Cruz Biotechnology) agarose beads were added and incubated for an additional 1 h at 4°C. After centrifugation (7000 ***g*** for 4 min at 4°C) and multiple washes, the immunoprecipitated complex was mixed with 2× SDS loading buffer and boiled for 5 min. The samples were then examined by Western blot analysis using a mouse monoclonal antibody against phosphorylated tyrosine (1:2000 dilution; Cell Signaling Technology).

### Cell surface biotinylation

Surface biotinylation experiments were performed in cultured rat granule neurons (5 days *in vitro*). After treatment, the cultured neurons were incubated with PBS containing 0.5–1.0 mg/ml sulfosuccinimidyl-6-[biotin-amido] hexanoate (Thermo Fisher Scientific) for 45 min on ice and were rinsed in ice-cold PBS containing 1 M glycine to quench the biotin reaction. The cultured neurons were lysed and then homogenized in modified radioimmune-precipitation assay buffer [50 mM Tris/HCl (pH 7.5), 150 mM NaCl, 1% Triton X-100, 10% glycerol, 0.5 mg/ml BSA, 1 mM PMSF, 1 μg/ml leupeptin, 1 μg/ml aprotinin and 1 μg/ml pepstatin]. The homogenates were centrifuged at 10000 ***g*** for 15 min at 4°C, and the supernatants were collected. The supernatants were incubated with 50 μl of NeutrAvidin–agarose (Thermo Fisher Scientific) overnight at 4°C and washed three times with radioimmune-precipitation assay buffer. The total and biotinylated surface proteins were detected using quantitative Western blots as described above.

### Data analysis

The results were analysed using a one-way ANOVA for com-parisons among multiple groups and using Student's *t* test for comparison between two samples. Results are means±S.E.M. For the electrophysiological experiments, data were collected from at least four different batches of neurons prepared on different dates, thereby minimizing the bias resulting from culture conditions. *P*<0.05 was considered statistically significant.

### Chemicals

Recombinant human GDF15 was purchased from Pepro Tech. We tested whether its biological activity was required for the GDF15-induced Kv2.1 expression by using heat-inactivated GDF15. After heat and radiation inactivation, the effect of GDF15 on Kv2.1 expression was diminished (results not shown). TTX, 4-AP, DRB (5,6-dichlorobenzimidazole riboside), CHX (cycloheximide), U0126, rapamycin, LY364947, PP2 and poly-L-lysine were purchased from Sigma. FBS, DMEM and antibiotic/antimycotic solution were purchased from Gibco Life Technologies.

## RESULTS

### GDF15 augments the *I*_K_ densities of CGNs in time- and dose-dependent manners at different developmental stages

**Figure 1:**
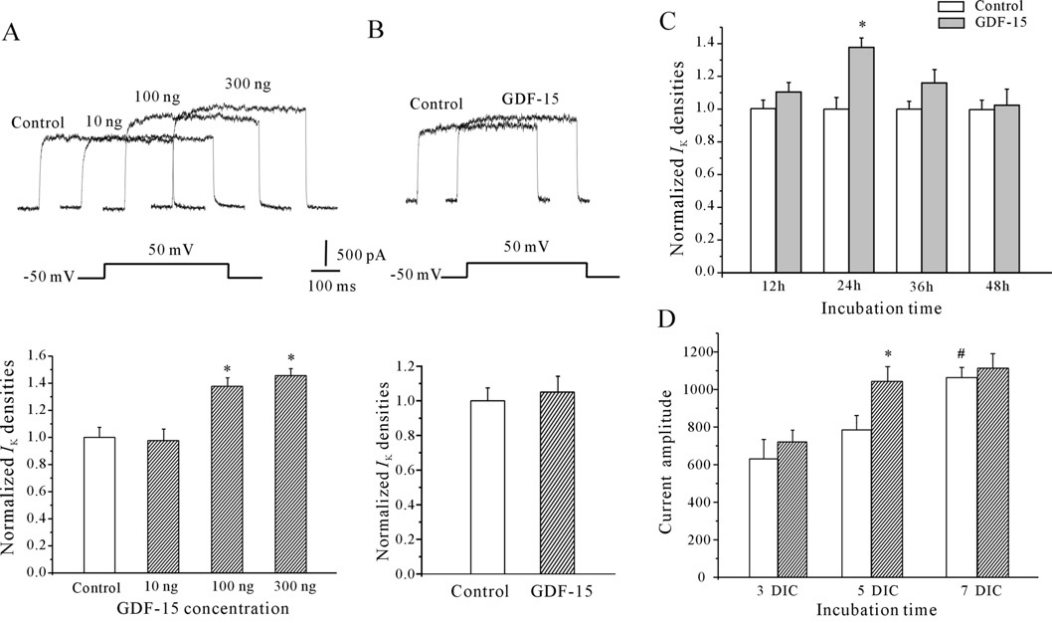
GDF15 enhanced the *I*_K_ densities in concentration- and time-dependent manners in CGNs (**A**) The *I*_K_ obtained from neurons maintained in control medium or medium with different concentrations of GDF15. The *I*_K_ was elicited by depolarizing pulses to +50 mV from a holding potential of −50 mV in the presence of 5 mM 4-AP. The upper panel shows representative recording samples and the lower panel shows statistical analyses (*n*=94). (**B**) The *I*_K_ obtained from neurons before and after acute application of the bath solution by a perfusion system (*n*=67). (**C**) The *I*_K_ densities in CGNs maintained for different incubation times in control medium or in medium containing 100 ng/ml GDF15 (*n*=74). (**D**) The *I*_K_ densities of the CGNs on different DIC maintained in control medium or in medium containing 100 ng/ml GDF15 for 24 h (*n*=88). Results are means±S.E.M. *P<0.05 compared with the control (without GDF15) determined using an unpaired Student's *t* test. #*P*<0.05 compared with 3 DIC.

CGNs contain two major voltage-dependent outward K^+^ currents *I*_K_ and *I*_A_. GDF15 treatment for 24 h failed to modulate the *I*_A_ in CGNs. Thus we examined the effect of GDF15 on *I*_K_ in the present study. *I*_K_ was evoked by a 100 ms depolarization to +50 mV from a holding potential of −50 mV in the presence of 5 mM 4-AP, which suppresses the *I*_A_ and permits better resolution of the *I*_K_. Incubation of the CGNs with GDF15 significantly enhanced the *I*_K_ densities in a concentration-dependent manner. The data obtained from 94 neurons showed that incubation of the CGNs with 10, 100 and 300 ng/ml GDF15 for 24 h increased the current densities by −2.2% (*n*=27), 37.7% (from 761.07±38.22 pA to 1048.40±40.25 pA; *n*=41; *P*>0.05) and 45.6% (*n*=26) respectively (Figures 1A and 1B). GDF15, however, did not affect the *I*_K_ amplitude of the CGNs when acutely applied to the bath solution using a perfusion system (Figure 1B). To address whether the GDF15-mediated *I*_K_ densities were time-dependent, the neurons were incubated with 100 ng/ml GDF15 for different time periods and the *I*_K_ densities were then determined. Although GDF15 was found to significantly augment the *I*_K_ densities by 37.7% at 24 h, incubation for 12, 36 and 48 h only produced slight increases of 7.4% (*n*=21), 14.3% (*n*=25) and 1.2% (*n*=28) respectively (Figure 1C). We also found that the effect of GDF15 on the induction of the *I*_K_ was dependent on the number of days that the CGNs had been in culture (Figure 1D). Together, these data indicate that incubating the granule neurons from 5 DIC with 100 ng/ml GDF15 for 24 h produced the most significant increase in the *I*_K_ density.

**Figure 2:**
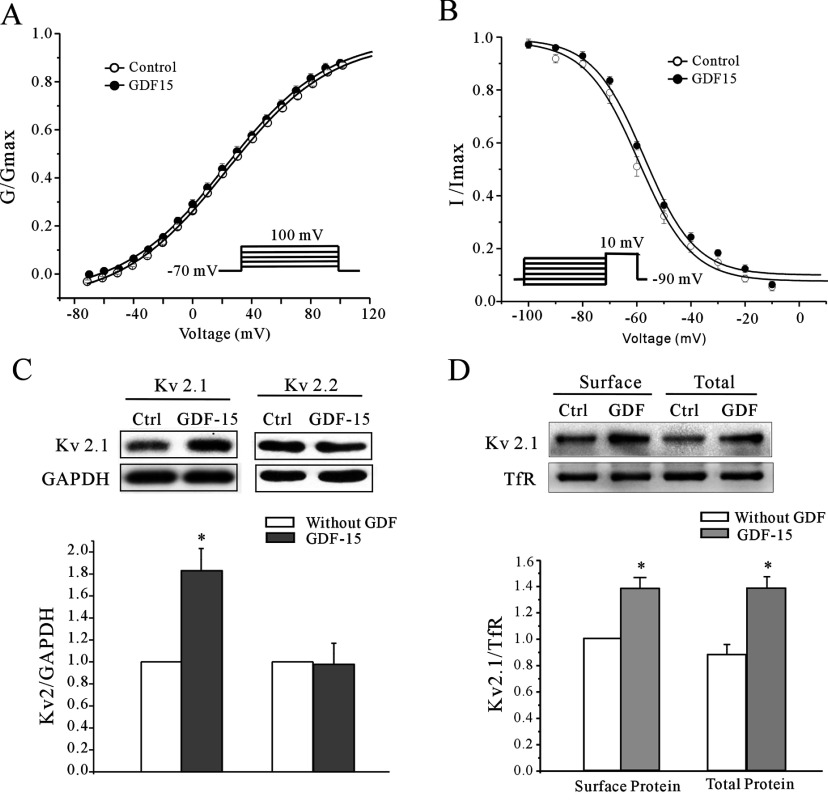
GDF15 did not alter the activation and inactivation properties of the *I*_K_ channels, but did up-regulate the Kv2.1 protein levels (**A**) Steady-state activation curves of the *I*_K_ obtained by plotting the normalized conductance as a function of command potential from control and GDF15-treated groups. The data points were fitted using the Boltzmann function. Results are means±S.E.M. (*n*=38 for the control group and *n*=42 for the GDF15-treated group). (**B**) Steady-state inactivation curves for the *I*_K_ obtained from control and GDF15-treated CGNs using the inactivation protocols (see the Experimental section). The data points were fitted using the Boltzmann function. Results are means±S.E.M. (*n*=21 for the control group and *n*=23 for the GDF15-treated group). (**C**) Western blot analysis showing the effect of 100 ng/ml GDF15 on the Kv2.1 and Kv2.2 expression levels in CGNs (*n*=5). The upper panel shows representative Western blots and the lower panel shows the statistical analysis. (**D**) Western blot analysis of membrane-bound Kv2.1 in CGNs incubated with GDF15 for 24 h (*n*=4). Transferrin (TFR) was used as a loading control. Endogenous GAPDH is shown as a cytoplasmic protein control. Results are means±S.E.M. *P<0.05 compared with the control (without GDF15) determined using an unpaired Student's *t* test. Ctrl, control.

We then examined whether the gating properties of the channels were altered, leading to the enhancement of the *I*_K_. We studied the voltage-dependent activation and deactivation properties of the *I*_K_ in control and GDF15-treated neurons. During steady-state activation experiments, the membrane potential was held at −70 mV, and the *I*_K_ was evoked by a 200 ms depolarizing pulse from a first pulse potential of −70 mV to +100 mV in 10 mV steps at 10 s intervals. The data were analysed using the equation *GK*=*I*_K_/(*V*_m_−*V*_rev_), where *GK* is the membrane K^+^ conductance, *V*_m_ is the membrane potential and *V*_rev_ is the reversal potential for K^+^. The *G*–*V* curve analysis indicated that GDF15 did not alter the half-maximal activation voltage of the *I*_K_ (Figure 2A) between the control (31.24±3.86 mV; *n*=17) and GDF15-treated (32.56±1.48 mV; *n*=23) neurons.

To study the steady-state inactivation of the *I*_K_, currents were elicited using 10 s conditioning pre-pulses from −90 mV to +10 mV before a 400 ms test pulse of +10 mV. After normalizing each current amplitude to the maximal current amplitude obtained from the −90 mV pre-pulse as a function of the conditioning pre-pulse potential and after fitting the normalization with the function *I*_K_/*I*_K_-max=1/(1+exp[(*V*_m_1/2−*V*_m_)/*k*]), we obtained an inactivation curve for the *I*_K_ and calculated the voltage at which the current amplitude was half-inactivated (*V*_h50_). The half-maximal deactivation voltage of the *I*_K_ in the control neurons (57.29±0.93 mV; *n*=15) was similar to that in the GDF15-treated neurons (57.55±2.44 mV; *n*=19) (Figure 2B). These results suggest that the GDF15-mediated enhancement of the *I*_K_ amplitudes was not associated with the modification of the *I*_K_ activation and inactivation properties.

**Figure 3:**
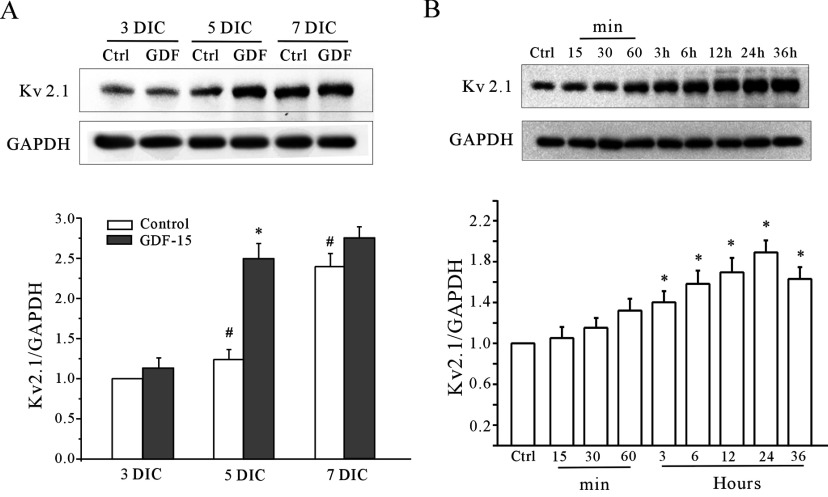
GDF15 increased the expression levels of Kv2.1 α-subunit in CGNs in a time-dependent manner (**A**) Western blot analysis showing the effect of 100 ng/ml GDF15 on Kv2.1 expression in CGNs after different DIC (*n*=3). *P<0.05 compared with the corresponding control (without GDF15) determined using an unpaired Student's *t* test. #*P*<0.05 compared with the 3 DIC group without GDF15 treatment. (**B**) The effect of 100 ng/ml GDF15 on the expression of Kv2.1 α-subunit protein in the CGNs incubated with GDF15 for 15 min to 36 h (*n*=4). *P<0.05 compared with the control (without GDF15) determined using an unpaired Student's *t* test. Ctrl, control.

Therefore we investigated whether the GDF15-mediated increase in the *I*_K_ amplitudes might be due to the up-regulation of channel expression levels. The primary α-subunits mediating the *I*_K_ in CGNs are Kv2.1 and Kv2.2 [19] and, therefore, Western blot analysis with specific antibodies against both Kv2.1 and Kv2.2 was performed to measure their expression levels after incubation with GDF15. The data indicate that Kv2.1 protein levels were significantly enhanced following incubation of the CGNs with GDF15 for 24 h at 5 DIC. However, there was no significant change in the total cellular protein expression levels of Kv2.2 (Figure 2C). Meanwhile, we investigated whether the membrane expression of Kv2.1 was affected in the neurons treated with GDF15. The surface proteins in the vehicle- or GDF15-treated neurons were isolated using the biotinylation assay, and Kv2.1 proteins were detected by immunoblot analysis with an anti-Kv2.1 antibody. As shown in Figure 2(D), the amount of Kv2.1 protein in the membrane fractions was greatly increased 24 h after GDF15 treatment (137.8±5.9% of the untreated level; *n*=4). The levels of transferrin 2, a subtype of transferrin, in the membrane fractions were not affected. Additionally, the kinetics of Kv2.1 expression induction paralleled the increase in the *I*_K_ amplitudes following GDF15 treatment (Figure 3). The CGNs from 5 DIC treated with 100 ng/ml GDF15 for 24 h produced the most significant enhancement of Kv2.1 protein expression (Figure 3). These findings suggest that GDF15 up-regulated the total cellular Kv2.1 expression as well as the associated *I*_K_ densities.

### GDF15 augments the total cellular levels of Kv2.1 through the dual regulation of protein translation and degradation

**Figure 4:**
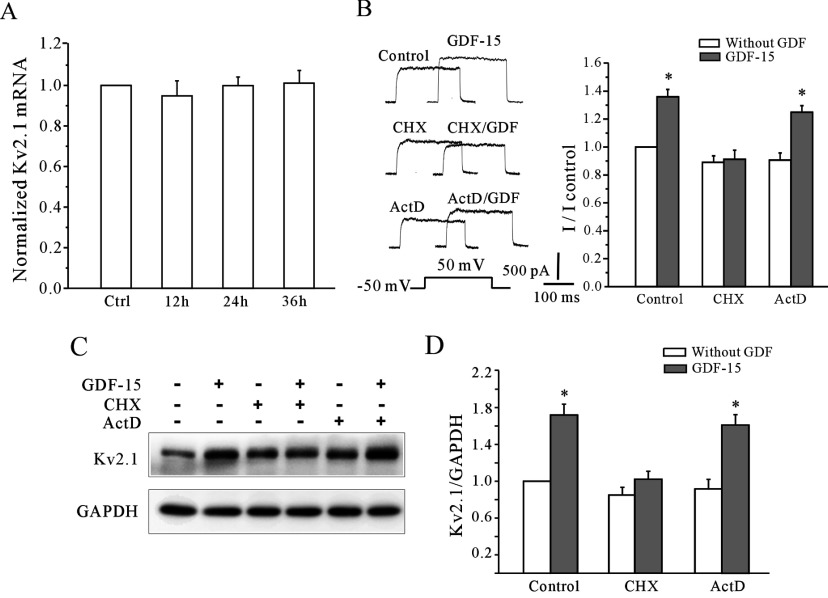
GDF15 elevated the *I*_K_ densities through the translational regulation of Kv2.1 α-subunit protein expression (**A**) Kv2.1 mRNA levels in the control and GDF15-treated CGNs from 12 to 36 h, as measured by qPCR analysis (*n*=4). (**B**) The effect of the transcriptional inhibitor actinomycin D (ActD) and the translational inhibitor CHX on the GDF15-induced up-regulation of the *I*_K_ densities. (**C** and **D**) Western blot and statistical analyses showing the effect of 10 μM actinomycin D and 10 μM CHX on the GDF15-induced up-regulation of Kv2.1 protein levels (*n*=4). Results are means±S.E.M. *P<0.05 compared with the corresponding control (without GDF15) determined using an unpaired Student's *t* test.

Gene expression changes in response to TGFβ are generally thought to take place at the transcriptional level [20,21] and, therefore, primers were used to measure the Kv2.1 mRNA expression levels by qPCR after incubation with or without GDF15. qPCR analysis revealed that the mRNA levels of the Kv2.1 α-subunit did not increase for incubation from 12 to 36 h (Figure 4A). The specific inhibitors actinomycin D and CHX were then used to further determine the roles of transcriptional and translational regulation in the GDF15-induced Kv2.1 expression. CGNs were pre-treated with 10 μM actinomycin D or 10 μM CHX for 30 min and then co-incubated with GDF15 for 24 h. The inhibitors alone had little effect on the *I*_K_ amplitude and Kv2.1 expression. The GDF15-induced increases in the *I*_K_ amplitudes were completely diminished by CHX treatment, but not by actinomycin D treatment (Figure 4B). Consistently, actinomycin D did not block the GDF15-induced augmentation of total cellular Kv2.1 expression (Figures 4C and 4D). Thus these data suggest a translational up-regulation of the Kv2.1 α-subunit by GDF15.

**Figure 5:**
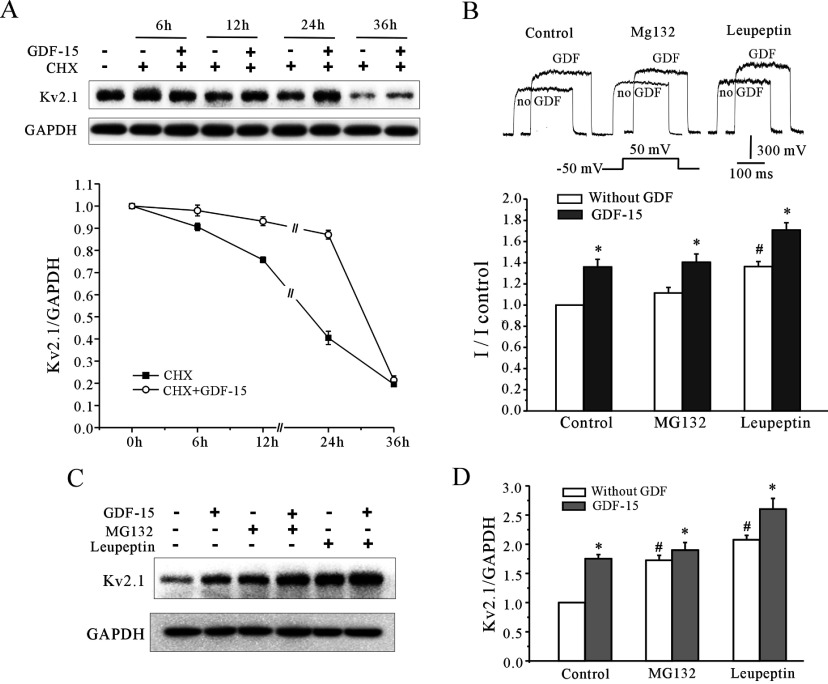
The protein degradation pathways contributed to the effects of GDF15 on the *I*_K_ densities and Kv2.1 protein levels (**A**) Stability of intracellular Kv2.1 as measured by CHX chase. The intracellular Kv2.1 level was assessed following treatment with 10 μM CHX from 6 to 36 h. A representative Western blot and statistical analysis are shown. (**B**) Effect of the protein degradation inhibitors 50 μM MG132 and 10 μM leupeptin, on the GDF15-induced increase in the *I*_K_ densities. (**C** and **D**) Representative Western blot and statistical analysis examining the effects of MG-132 and leupeptin on the GDF15-induced up-regulation of Kv2.1 protein levels (*n*=3). Results are means±S.E.M. *P<0.05 compared with the corresponding control (without GDF15) determined using an unpaired Student's *t* test. #*P*<0.05 compared with the control without MG132 and leupeptin.

In addition to the translation rate, the intracellular protein content also depends on the degradation rate. To determine if the augmentation of Kv2.1 protein levels was due to reduced degradation, CHX chase assays were performed [22]. Cultured CGNs were incubated with 10 μg/ml CHX in the culture medium for 6, 12, 24 or 36 h with or without 100 ng/ml GDF15 and were then harvested for Western blot analysis. The control cells were treated with DMSO, the vehicle used for drug preparation. As shown in Figure 5(A), Kv2.1 has a long half-life (~20 h) in the CGNs. When GDF15 was present in the culture medium, this half-life increased to ~30 h. Moreover, the administration of GDF15 protected Kv2.1 from degradation within 12 h compared with the control group (Figure 5A).

The UPS (ubiquitin–proteasome system) is responsible for degrading most of the intracellular soluble proteins, and the lysosomes degrade most membrane and endocytosed proteins. Consequently, we explored whether these two pathways assisted GDF15 in regulating the *I*_K_ amplitude and the amount of Kv2.1 using specific inhibitors of these pathways. We pre-incubated CGNs with 50 μM MG-132 (a proteasome inhibitor) or 10 μM leupeptin (a lysosome inhibitor) for 30 min before treatment with GDF15. MG-132 alone did not mimic the GDF15-induced increase in the *I*_K_ amplitude after incubation for 24 h, and it actually augmented the levels of total cellular Kv2.1 protein, similar to GDF15 (Figures 5B–5D). Compared with the effects of MG-132 on the *I*_K_ amplitude and Kv2.1 protein levels, leupeptin completely mimicked the effect of GDF15. Taken together, these results suggest that translational regulation co-operates with the lysosome-dependent protein degradation pathway to mediate the GDF15-induced augmentation of Kv2.1 protein expression and the *I*_K_ amplitude.

### GDF15 induces Akt/mTOR and ERK1/2 phosphorylation

**Figure 6:**
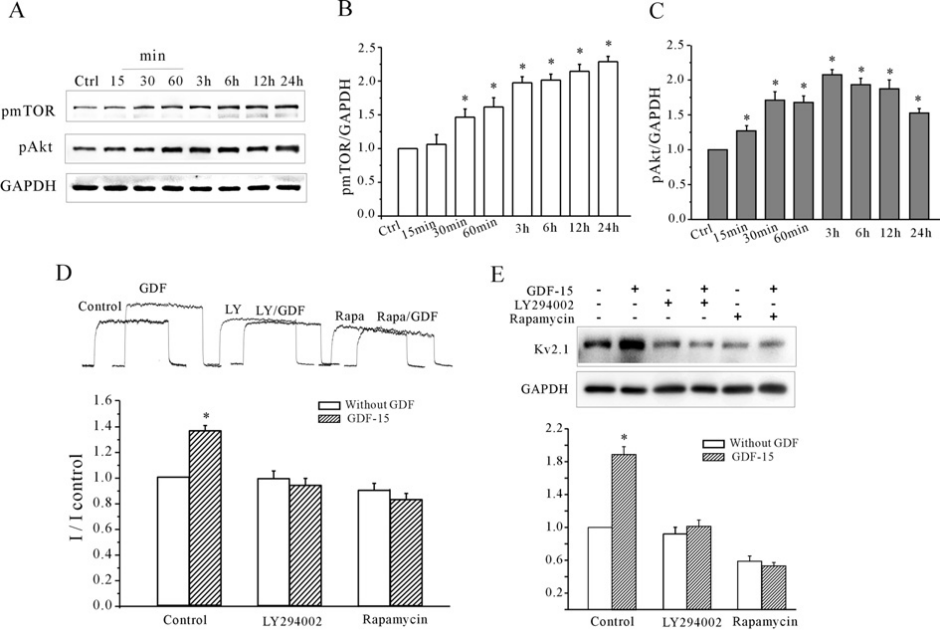
The GDF15-induced *I*_K_ densities and increased expression of the Kv2.1 α-subunit were dependent on the Akt/mTOR signalling pathway (**A**) Representative Western blot analysis showing the levels of activated Akt (pAkt) and mTOR (pmTOR) in CGNs after incubation with 100 ng/ml GDF15 for 15 min to 24 h. (**B** and **C**) Quantification of p-Akt and p-mTOR in CGNs after incubation with 100 ng/ml GDF15 for 15 min to 24 h (*n*=5). (**D**) The effects of the Akt inhibitor LY294002 (LY) and the mTOR inhibitor rapamycin (Rapa) on the GDF15-induced up-regulation of the *I*_K_ densities (*n*=4). (**E**) The effects of LY294002 and rapamycin on the GDF15-induced up-regulation of Kv2.1 protein levels (*n*=4). Results are means±S.E.M. *P<0.05 compared with the corresponding control (without GDF15) determined using an unpaired Student's *t* test.

Because our data indicated that GDF15 increased Kv2.1 protein synthesis and prevented its degradation, we first tried to determine whether the Akt/mTOR pathway, which has been reported to affect protein synthesis and CGN mutation [17,23], was activated by GDF15. After GDF15 treatment, the phosphorylation levels of mTOR at Ser^2448^ (p-mTOR) began to increase at 30 min (146.4±5.8%) and then continued to increase to 228.7±3.6% by 24 h. For Akt phosphorylated at Ser^473^ (p-Akt), its phosphorylation level significantly changed by 26.9±1.6% after 15 min, and phosphorylation reached its peak after 3 h (*n*=4) (Figures 6A–6C). The requirement of the Akt/mTOR pathway for the GDF15-induced up-regulation of the *I*_K_ density and Kv2.1 α-subunit expression was confirmed using pharmacological inhibitors. Figures 6(D) and 6(E) show that blockade of PI3K (phosphoinositide 3-kinase)/Akt/mTOR activity by LY294002 or rapamycin treatment prevented the GDF15-mediated increase in the *I*_K_ density and the increase in Kv2.1 protein expression. In the presence of LY294002 or rapamycin, the GDF15-induced increase in the *I*_K_ density was reduced (Figure 6D) from 37.5% to −0.11% (*n*=36; *P*<0.05) or from 36.4% to −17.6% (*n*=29; *P*<0.05) respectively. Similarly, LY294002 and rapamycin decreased the expression levels of the Kv2.1 α-subunit protein (Figure 6E) to 0.9±1.9% (*n*=4) and −47.3±2.2% (*n*=4) respectively. These data indicate that the mTOR pathway is also required for the GDF15-stimulated up-regulation of the *I*_K_ density and increase in Kv2.1 α-subunit expression.

**Figure 7:**
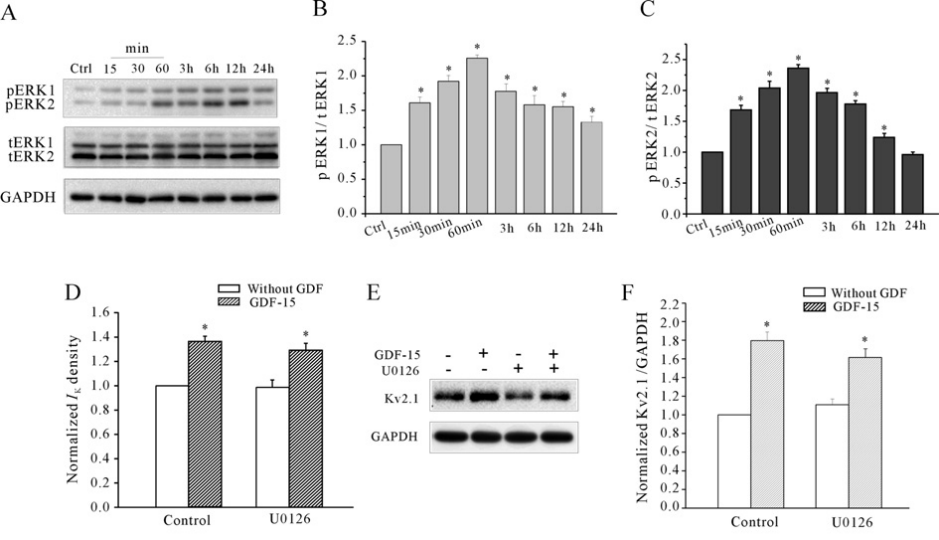
GDF15 stimulated activation of the MAPK/ERK1/2 pathway (**A**) Western blot analysis showing the levels of phosphorylated ERK1 (pERK1) and phosphorylated ERK2 (pERK2) in CGNs incubated with 100 ng/ml GDF15 for 15 min to 36 h. (**B** and **C**) Statistical analysis of p-ERK1 (*n*=3) and pERK2 (*n*=3) in CGNs incubated with 100 ng/ml GDF15 for 15 min to 36 h. (**D**) The effect of the MEK inhibitor U0126 on the GDF15-induced up-regulation of the *I*_K_ densities. (**E** and **F**) The effect of U0126 on the GDF15-induced up-regulation of Kv2.1 protein levels. Results are means±S.E.M. *P<0.05 compared with the corresponding control (without GDF15) determined using an unpaired Student's *t* test. Ctrl, control; tERK, total ERK.

In addition to Akt/mTOR signalling, we found that GDF15 induced the parallel activation of the MAPK (mitogen-activated protein kinase) pathway. After GDF15 treatment, the phosphorylation levels of ERK1 and ERK2 (p-ERK1 and p-ERK2) were significantly increased, reaching the maximal levels of 225.7% and 235.9% (*n*=3) respectively at 60 min (Figures 7A–7C). Surprisingly, inhibiting MEK (MAPK/ERK kinase) with U0126 did not block the GDF15-stimulated *I*_K_ density and Kv2.1 protein increases (Figures 7D–7F). These data indicate that both the Akt/mTOR and MAPK/ERK pathways are activated by GDF15, but the MAPK/ERK pathway is not required for the GDF15-mediated up-regulation of the *I*_K_ densities and increases in Kv2.1 α-subunit expression.

### TGFβR2 is required for the effect of GDF15 on Kv2.1 expression and GDF15 signalling through the Akt/mTOR and MAPK/ERK pathways

**Figure 8:**
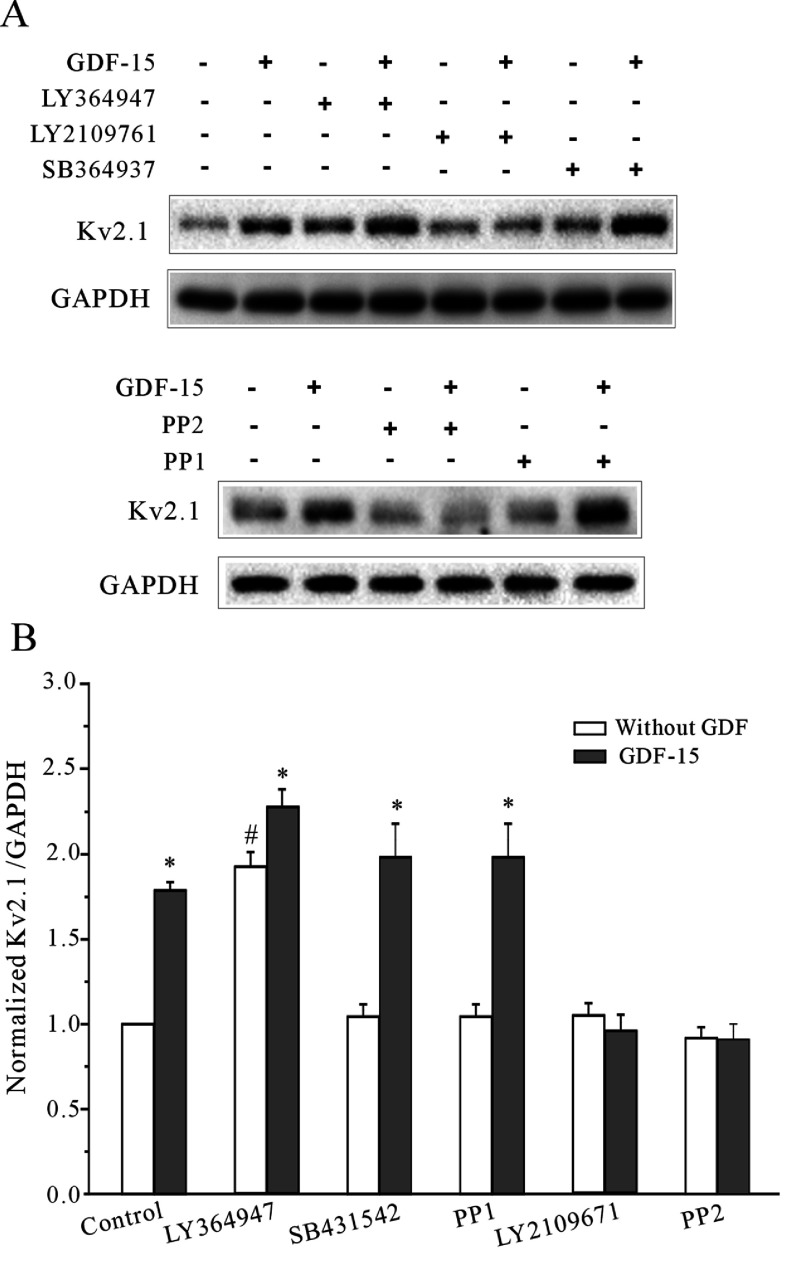
The effects of TGFβR1 inhibitors and TGFβR1/TGFβR2 dual inhibitors on GDF15-induced Kv2.1 expression (**A**) Representative Western blot showing the effects of the TGFβR1 inhibitors LY364947, SB431542 and PP1 and the TGFβR1/TGFβR2 dual inhibitors PP2 and LY2109761 on the GDF15-induced up-regulation of Kv2.1 protein levels (*n*=3). (**B**) Statistical analysis showing the effects of LY364947, SB431542, PP1, PP2 and LY2109761 on the GDF15-induced up-regulation of Kv2.1 protein levels (*n*=3). Results are means±S.E.M. *P<0.05 and #*P*<0.05 compared with the corresponding control (without GDF15) determined using an unpaired Student's *t* test. #*P*<0.05 compared with control without TGFβR1 or TGFβR1/TGFβR2 dual inhibitors.

Previous studies have suggested that successively activating the TGFβR1–TGFβR2 heterodimer, a cell surface receptor complex, is needed for the TGFβ subfamily to achieve cross-membrane signalling [24,25]. Thus we used TGFβR1 (ALK5) inhibitors (LY364947, SB431542 and PP1) and TGFβR1/TGFβR2 dual inhibitors (PP2 and LY2109761) to determine whether the effect of GDF15 on Kv2.1 expression was associated with the TGFβR1–TGFβR2 heterodimer complex [26]. Inhibition of the TGFβR1 kinase using 200 μM LY364947, 10 μM SB431542 or 10 μM PP1 did not reduce the GDF15-induced augmentation of Kv2.1 α-subunit expression. In contrast, inhibition of TGFβR1/TGFβR2 by PP2 or LY2109761 significantly blocked the GDF15-induced augmentation of Kv2.1 α-subunit expression (Figure 8).

**Figure 9:**
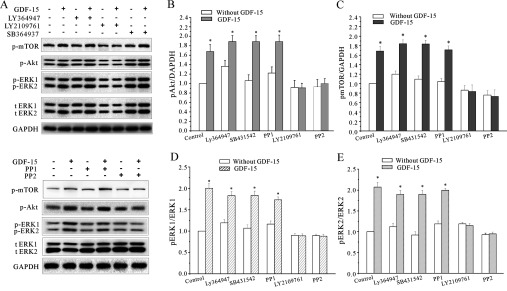
The effects of the TGFβR1 or TGFβR1/TGFβR2 dual inhibitors on GDF15 signalling to the Akt/mTOR and MAPK/ERK pathways (**A**) Western blot analysis showing the effects of the TGFβR1 inhibitors LY364947, SB431542 and PP1, and the TGFβR1/TGFβR2 dual inhibitors PP2 and LY2109761 on the GDF15-stimulated phosphorylation of mTOR, Akt and ERK1/2 (*n*=3). (**B**–**E**) Statistical analysis of the effects of the TGFβR1 or TGFβR1/TGFβR2 dual inhibitors on the GDF15-stimulated p-Akt, p-mTOR, p-ERK1 and p-ERK2 (*n*=3). Results are means±S.E.M. *P<0.05 compared with the corresponding control (without GDF15) determined using an unpaired Student's *t* test. t ERK, total ERK.

We then observed the effects of these five inhibitors on the GDF15-induced activation of both the Akt/mTOR and MAPK/ERK pathways. The inhibitors were pre-incubated with the CGNs for 30 min, and GDF15 was then added to the medium for 30 min. In the CGNs treated with the TGFβR1 inhibitors, the GDF15-induced phosphorylation of Akt, mTOR and ERK1/2 was not changed (Figure 9A). In contrast, PP2 and LY2109761 were able to eliminate the GDF15-induced phosphorylation of mTOR, ERK1/2 and Akt (Figure 9).

**Figure 10:**
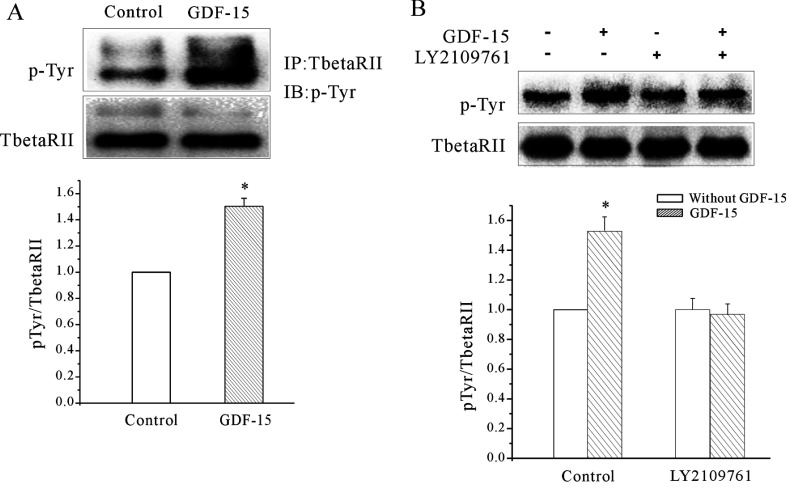
GDF15 induced the tyrosine phosphorylation of TGFβR2 (**A**) Immunoprecipitation (IP) assays showing that GDF15 induced the tyrosine phosphorylation (p-Tyr) of TGFβR2 in the CGN lysates (*n*=3). *P<0.05 compared with the corresponding control (without GDF15) determined using an unpaired Student's *t* test. (**B**) Immunoprecipitation assays showing that pre-treatment with LY2109761 inhibited the GDF15-induced tyrosine phosphorylation of TGFβR2 (*n*=3). *P<0.05 compared with the corresponding control (without GDF15) determined using an unpaired Student's *t* test. IB, immunoblot. IP, immunoprecipitation.

Because Bandyopadhyay et al. [26] suggested that TGFβR2 alone is able to mediate TGFβ signalling to ERK1/2 without the participation of TGFβR1 and that phosphorylation of Tyr^284^ in TGFβR2 is associated with the activation of non-Smad signalling pathways [27], we analysed the GDF15-elicited TGFβR2 activation of tyrosine residues by immunoprecipitation. The immunoprecipitation assays showed a significantly increased amount of phosphorylated tyrosine residues on TGFβR2 after GDF15 treatment for 30 min (Figure 10A). Meanwhile, pre-treatment of the CGNs with 5 μM LY2109761 for 30 min could efficiently block the phosphorylation of the tyrosine residues on TGFβR2 after exposure to GDF15 for 30 min. (Figure 10B)

## DISCUSSION

GDF15, also known as MIC-1, is a novel member of the TGFβ superfamily and is known to plays multiple roles in the processes of neural protection, regeneration and axonal elongation [5,7,9,13,28]. However, the receptor for GDF15 and its downstream effector signalling pathways have been poorly characterized. In the present study, for the first time, we show that GDF15 may activate the TGFβR2 and PI3K/Akt/mTOR signalling pathways to increase the *I*_K_ amplitude, as well as the expression of Kv2.1 in CGNs, which may be associated with a developmental function.

The *I*_K_ is one of the most ubiquitously expressed voltage-gated K^+^ channels and plays many diverse physiological roles. Kv2.1 is a major component of the *I*_K_ in the central nervous system [19]. Previous studies using cultured CGNs showed that enhancement of the *I*_K_ was associated with the apoptosis, migration or maturation of CGNs, depending on their normal development state or abnormal apoptosis stimulation [14,15]. Indeed, the *I*_K_ amplitude and the expression of Kv2.1 in normal cultured CGNs increased with the duration of the culture period [29]. We observed a concomitant increase in the *I*_K_ and Kv2.1 expression from 3 to 7 DIC (Figures 1D and 3A), which reached the maximum at 6–7 DIC [18]. Notably, the effect of GDF15 on the increase of the *I*_K_ and Kv2.1 expression was significant in 5 DIC CGNs, and after GDF15 treatment the levels of the *I*_K_ and Kv2.1 in the 5 DIC CGNs were similar to those of 7 DIC CGNs (Figures 1D and 3A). It is probable that 5 DIC is the critical period for Kv2.1 and CGN maturation and, thus, these cells are sensitive to GDF15. Once the CGNs have matured or Kv2.1 expression reaches its maximum plateau, the effect of GDF15 on Kv2.1 is no longer significant. In addition to Kv2.1, the expression levels of GABA_α6_ (γ-aminobutyric acid α6) receptor and p-CREB (cAMP-response-element-binding protein), two typical markers of CGN development, were also enhanced by GDF15 treatment (results not shown). Taken together, our observations suggest that the effect of GDF15 on Kv2.1 expression may be developmentally regulated and associated with neuronal maturation. Because multiple factors are associated with the developmental regulation of CGNs, further investigations are needed to confirm this speculation.

The activity of mTOR, a known promoter of cell growth, is modulated in response to various stimuli, such as neurotrophic factors and mitogenic hormones, to achieve transcriptional and translational control [23,30–32]. In CGNs, the Akt/mTOR pathway, which is activated by neuritin, is known to up-regulate Kv4.2 gene transcription, resulting in an increase in the *I*_A_ densities, which is also associated with the development and maturation of CGNs [17]. In the present study, we showed that the mTOR-specific inhibitor rapamycin blocks the GDF15-mediated induction of Kv2.1 expression, as well as the subsequent increase in the *I*_K_ (Figure 6). Thus GDF15 controls Kv2.1 gene expression through the mTOR pathway. Although few, if any, studies have reported a GDF-mediated mTOR signalling pathway in the central and peripheral nervous systems, the results of the present study are consistent with investigations in mouse fibroblasts and renal fibrogenesis of obstructive nephropathy, in which TGFβ was shown to be coupled with the PI3K/Akt/mTOR pathway [33,34]. These data indicate that the non-canonical TGFβ pathways are activated during GDF15 modulation of CGN development.

A balance between the synthesis and degradation of cellular proteins is of key importance in the regulation of protein levels, including ion channel proteins [35]. Interestingly, in addition to improving translation, GDF15 enhanced the *I*_K_ by inhibiting the degradation of Kv2.1. Protein degradation occurs via targeting of the protein by ubiquitination, and subsequent degradation occurs in the cellular proteasome or via lysosomal catalysis, which is associated with cellular autophagy [36]. Na^+^ and Ca^2+^ channel proteins, such as Nav1.5, Cav2.2 and Cav1.2 of the L-type channels, are known to be degraded mainly through ubiquitination-mediated proteasomal degradation [37–40]. In contrast, no single K^+^ channel protein degradation pathway seems to exist, probably due to the diversity in the structure and composition of the subunits. In the present study, pharmacologically blocking the proteasomal pathway or the lysosomal pathway increased total Kv2.1 protein levels, but only blocking the lysosomal degradation pathway resulted in increased the *I*_K_ densities. This result was similar to that of previous studies using a patch clamp to detect *K*_ATP_ and Kir2.1 channel expression at the plasma membrane [41,42]. Moreover, the GDF15-induced increases in Kv2.1 expression and the *I*_K_ densities were more significant in the presence of leupeptin, suggesting that the lysosomal-dependent degradation of Kv2.1 is more important for the breakdown of the functional *I*_K_ channels on the plasma membrane (Figure 5). Little is known about the ability of the K^+^ channels, in particular Kv2.1, to undergo multiple rounds of internalization and recycling. On the basis of the results of the present study and on those of Weigel et al. [43], who suggested that Kv2.1 proteins in CGNs are long-lived on the surface of mammalian cells and that autophagy is the major cellular pathway for the degradation of long-lived proteins and cytoplasmic organelles, we speculate that the GDF15-induced Akt/mTOR pathway negatively modulates the lysosomal degradation of Kv2.1, similar to how mTOR signalling is the principal negative regulator of macroautophagy [36].

In addition to the Akt/mTOR pathway, the MAPK/ERK pathway was also activated by GDF15 exposure in the present study (Figure 7), similar to the results reported for hypothalamic neurons and dermal cells [26,44]. A previous study indicated that ERK activation could dually regulate the K^+^ channel subunits at the transcriptional and post-translational levels [45]. ERK could directly phosphorylate the subunits of the ion channels, which were characterized by changes in the gating properties of the channels, such as upon acute regulation of the *I*_A_ by growth factors [46,47]. However, the results of the present study show that GDF15 neither alters the gating properties of the *I*_K_ nor has an acute effect on *I*_K_ amplitude. Moreover, blocking the ERK pathway does not eliminate the GDF15-induced effect on Kv2.1 expression. These data indicate that activating the ERK pathway is not necessary for the GDF15-induced increase in Kv2.1 expression and the *I*_K_ density. Subramaniam et al. [13] showed that GDF15 protected CGNs from low K^+^-induced cell death by activating the PI3K/Akt pathway and by inhibiting the endogenously active ERK. These opposite effects might be due to the different culture conditions of the CGNs. It is quite possible that GDF15 activates the ERK pathway in CGNs under normal culture conditions, but eliminates ERK activation induced by low K^+^-induced apoptosis stimulation. Bandyopadhyay et al. [26] reported that TGFβ selectively activated ERK1/2 in dermal cells and inhibited ERK1/2 in epidermal cells. They concluded that these opposite effects correlated with the distinct expression levels of TGFβR2, which were 7–18-fold higher in the dermal cells than in the epidermal cells. Whether these opposite effects of GDF15 on the ERK levels in CGNs were also due to the distinct expression of TGFβR2 induced by the different culture conditions warrants further investigation.

Although previous studies implied that GDF15, a member of the TGFβ subfamily, induces a signal transduction pathway very similar to that of the TGFβ family, only a few studies have identified the receptor through which GDF15 transduces its signal [44,48,49]. Previous studies have suggested that the TGFβ subfamily signals at the membrane are transmitted via a cell surface receptor complex, the TGFβR2–TGFβR1 heterodimer, thereby achieving cross-membrane signalling into the interior of the cell [24,25]. The post-receptor signalling is divided into the R-Smad (receptor-regulated Smad)-dependent pathways, such as Smad2/3, and the R-Smad-independent pathways, such as MEKK1 (MEK kinase 1), ERK1/2, p38/MAPK and PI3K [20,21]. In the present study, GDF15 activates both ERK and mTOR signalling, and blocking mTOR signalling and inhibiting tyrosine phosphorylation on TGFβR2 eliminated the GDF15-induced effects on Kv2.1 and the *I*_K_ densities, suggesting that an R-Smad-independent pathway might be involved. More intriguingly, blocking TGFβR1 by treatment with LY364947, SB431542 or PP1 could not abolish the GDF15-induced effects on the CGNs, including the activation of the ERK1/2 and Akt/mTOR pathways and increased Kv2.1 expression. In contrast, the inhibition of TGFβR1/TGFβR2 by treatment with PP2 or LY2109761 significantly blocked the GDF15-induced augmentation of Kv2.1 α-subunit expression and the activation of the ERK1/2 and Akt/mTOR pathways (Figures 8 and 9). Moreover, direct tyrosine phosphorylation of TGFβR2 elicited by GDF15 was also observed in the CGNs (Figure 10). Taken together, these data suggest that TGFβR1 activation does not appear to be important for the effect of GDF15 on CGNs. However, although there is some evidence from human skin cells and breast cancer cells that indicates that TGFβR2 alone is able to mediate TGFβ signalling to ERK1/2 without participation of TGFβR1 [26,27], whether TGFβR2 alone is also able to mediate GDF15 signalling to ERK1/2 and mTOR activation in CGNs has yet to be determined.

Our previous study reported that TGFβ1 increases the *I*_K_ amplitudes by up-regulating Kv2.1 expression, which requires PKA (protein kinase A) activity [12]. However, although the GDF15-induced increase in the *I*_K_ densities and Kv2.1 expression was similar to that caused by TGFβ1, the mechanism and biological significance seem quite different. We observed that the *I*_K_ densities and Kv2.1 expression induced by TGFβ_1_ are sensitive to the narrow time-window of 7–8 DIC, in which the *I*_K_ properties are close to those of mature CGNs [50], but the GDF15-induced effect on the *I*_K_ density and Kv2.1 expression only occur at 4–5 DIC (Figure 1), when the neurons were in an immature state. Mechanistically, TGFβ1 acts at the translational level, rather than by blocking degradation, to enhance Kv2.1 protein expression and the *I*_K_ density, and this activity was associated with the cAMP/PKA pathway rather than with the Akt/mTOR pathway. These differences between TGFβ1 and GDF15 in the temporally co-ordinated manner and post-receptor signalling of Kv2.1 expression might be related to the cell surface expression profiles of both TGFβR1 and TGFβR2 or to distinct oligomeric interactions that occur during signalling. These observations are in accordance with the hypothesis postulated by Ehrlich et al. [51,52], who indicated that different members of the TGFβ subfamily played distinct functions through distinct oligomeric interactions during signalling, and these interactions were dependent on the cell type, maturation state and developmental stage of the cells.

In conclusion, we showed that GDF15 potentiates the *I*_K_ densities by increasing the expression of Kv2.1 in CGNs. The effect of GDF15 on Kv2.1 expression might be channelled through TGFβR2 to activate the Akt/mTOR pathway. Thus the GDF15–TGFβR2–Kv2.1–*I*_K_ axis may play an important role in neuronal development and the maturation of CGNs. However, due to technical limitations, certain interesting questions, such as how phosphorylated TGFβR2 activates the Akt/mTOR pathway and why the time-window of GDF15 responsiveness is extremely narrow, cannot be completely explained in the present study. The present study could serve as a foundation to guide further in-depth studies of GDF15 function and mechanism.
